# Granular vortex spin-torque nano oscillator for reservoir computing

**DOI:** 10.1038/s41598-023-43923-z

**Published:** 2023-10-04

**Authors:** S. Shreya, A. S. Jenkins, Y. Rezaeiyan, R. Li, T. Böhnert, L. Benetti, R. Ferreira, F. Moradi, H. Farkhani

**Affiliations:** 1 Electrical and Computer Engineering Department, Aarhus University, 8200 Aarhus, Denmark; 2 International Iberian Nanotechnology Laboratory (INL), Braga, Portugal

**Keywords:** Nanoscale devices, Electrical and electronic engineering

## Abstract

In this paper, we investigate the granularity in the free layer of the magnetic tunnel junctions (MTJ) and its potential to function as a reservoir for reservoir computing where grains act as oscillatory neurons while the device is in the vortex state. The input of the reservoir is applied in the form of a magnetic field which can pin the vortex core into different grains of the device in the magnetic vortex state. The oscillation frequency and MTJ resistance vary across different grains in a non-linear fashion making them great candidates to be served as the reservoir's outputs for classification objectives. Hence, we propose an experimentally validated area-efficient single granular vortex spin-torque nano oscillator (GV-STNO) device in which pinning sites work as random reservoirs that can emulate neuronal functions. We harness the nonlinear oscillation frequency and resistance exhibited by the vortex core granular pinning of the GV-STNO reservoir computing system to demonstrate waveform classification.

## Introduction

Recent advances in neuroscience, combined with the development of nanoscale electronic devices, have resulted in a great deal of interest in developing brain-like computing hardware utilizing nanoscale memory components as neuronal and synaptic elements^1^. Among them, Reservoir Computing (RC) which is a type of recurrent neural network (RNN) is a promising candidate, with interconnected neurons that transform the input data into a rich temporal outcome^2^. Time-dependent input signals result in nonlinearly varying outcomes which also depend on previous data or ‘Echo’, making these frameworks well-suited to time-series information handling, processing, and determining assignments^3^. The crucial element of RC is randomly connected reservoirs that transform the sequential input data into high-dimensional features including spatiotemporal patterns such that the features of the inputs can be efficiently read out. Simple classification is then performed in the readout layer. In contrast to other types of artificial neural networks (ANN), in RC, the weights of the reservoir (*W*_*R*_) are not trained whereas only the readout weights (*W*_*out*_) are trained with a simple training algorithm, leading to a reduction of the training cost significantly. In general, a physical phenomenon that satisfies the main characteristics of a reservoir which are nonlinearity to separate inputs of different classes but not become unstable with noise^4^, and short-term memory (STM)^5^ to ensure that the reservoir state only depends on recent-past inputs (with a time scale longer than meaningful temporal correlations of the input). Recently, different physical systems and devices have been proposed for realizing the reservoir such as fluidic^6^ and biological^7^, electronic circuits^8^, photonic^9^, and spintronic^10–15^.

Among different physical systems and devices for realizing a reservoir, spintronic nano-devices are promising candidates due to their unique features such as high-speed dynamics, power efficiency, high density, and CMOS compatibility. In terms of accuracy, they have some other dynamic features such as STM, recurrence behaviors, and nonlinearity, which make them very attractive for the realization of the reservoir^3,15–17^. Magnetic Tunnel Junctions (MTJs) are the fundamental building block for a range of emerging technologies, including fully developed ideas such as magnetic memories^18^ and sensors^19^, as well as more fundamental concepts such as energy harvesters^20,21^, microwave frequency generation^22^, and neuromorphic computing systems (NCS)^23,24^. Microwave frequency can be generated using spintronic devices commonly known as spin-torque nano oscillators (STNOs) that can emulate brain-like neural functionality at the nanoscale^23^. In particular, vortex-STNOs, where the free layer of the MTJ is the non-homogeneous magnetic texture known as a magnetic vortex, are very suitable for neuromorphic computing owing to their high emission power, narrow linewidth, and mid-to-high microwave frequency generation properties^22,25^. Magnetic spin textures such as skyrmions and magnetic vortices have recently been studied for unconventional computing due to their small size, memory capacity, and magnetics dynamics^10,26^. A few works have been reported wherein an array of STNOs are used to develop a RC system^10,13^. However, the complex arrayed fabrication and the low output power of the STNOs have made it difficult for STNO implementations as RC system^10–14,27^. Skyrmionic reservoirs are reported to overcome the design complexity^15,27^, however, the small anisotropy magnetoresistance (AMR)^12^ is seen as their biggest challenge when it comes to reading out the output signal. Hence, in this work, we propose using a single granular vortex STNO (GV-STNO) to be used as an RC system. The different pinning sites caused by the grains present in the free layer (FL) function as randomly distributed reservoirs providing distinct output frequency (*f*_*out*_) and the device resistance (*R*_*MTJ*_) for an applied magnetic field input to the system. Moreover, very few works are reported studying the effect of granularity in different layers^28–32^, however, here we present the use of a single device to be used for RC system and its application. By analyzing the magnetization components, i.e., *f*_*out*_ and/or *R*_*MTJ*_, RC capability can be achieved for various applications (such as pattern recognition, image recognition, classification, and so on).

The main contributions of this work are (1) the study of the granular effect on the vortex core dynamics of the FL of MTJ, (2) the development of the novel concept of reservoir computing (attaining key features- nonlinearity and STM) using single MTJ device wherein the grains work as the reservoir neurons, and (3) the demonstration of waveform classification as an application of RC.

### Granular vortex spin-torque nano oscillator

**Figure 1:**
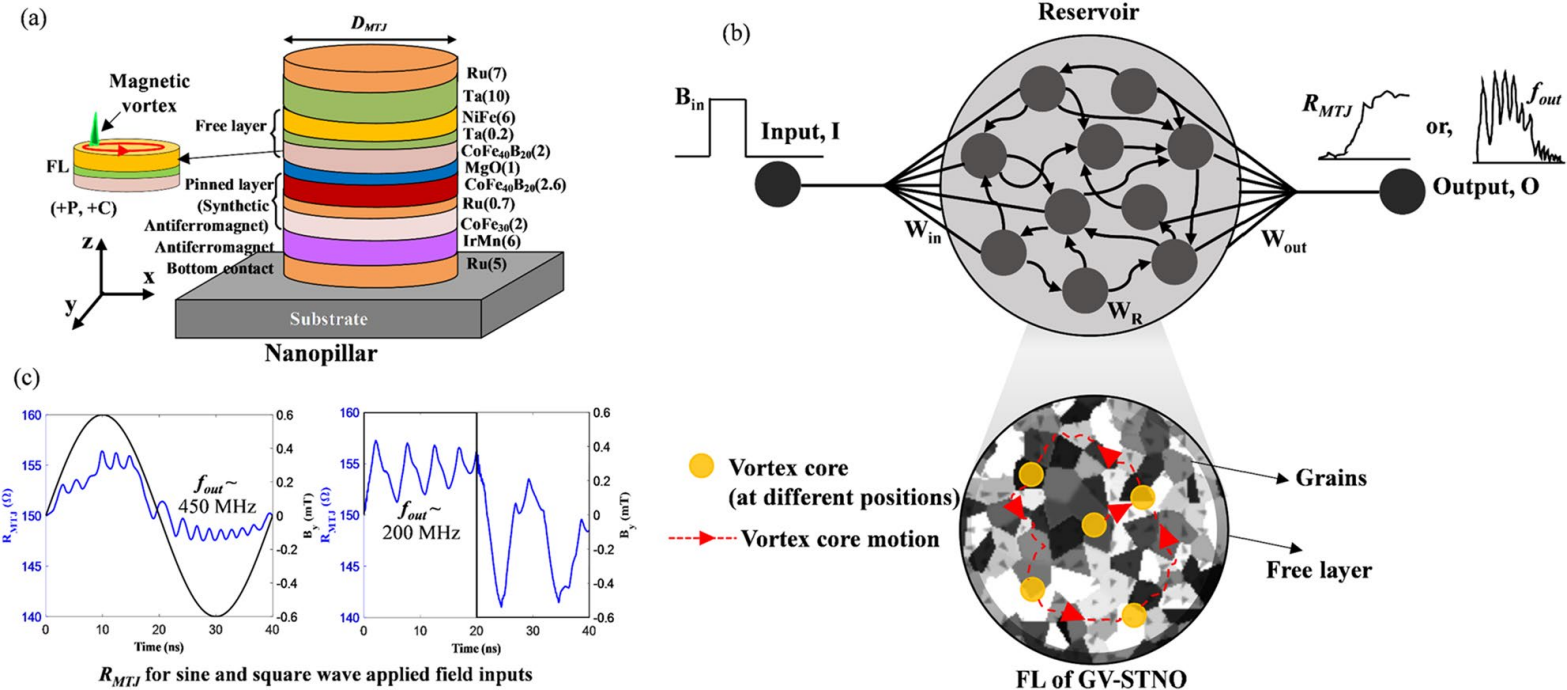
Concept of reservoir computing using the granular vortex spin torque nano oscillator, the vortex core formed starts to oscillate and due to the grain effects, it may generate different output frequencies observed from the magnetization components which is related to the resistance of the device, (**a**) GV-STNO nanopillar schematic, (**b**) RC system using single GV-STNO device and how grains can work as different reservoir neurons with variable weights, *W*_*R*_, and (**c**) neuron output *W*_*out*_ in form of *R*_*MTJ*_ and *f*_*out*_ of GV-STNO.

**Figure 2:**
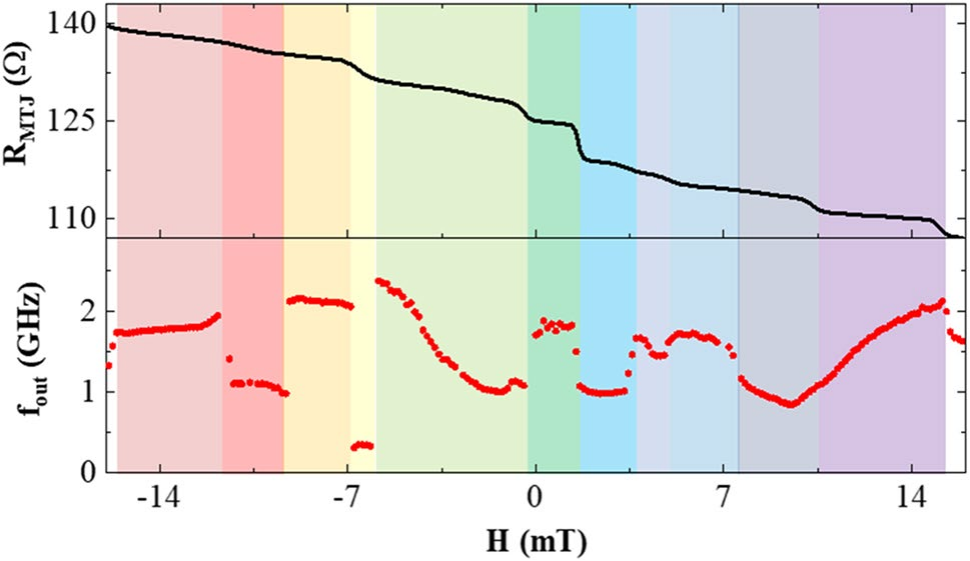
Experimental measurement of granular effect in the sample showing nonlinearity in *f*_*out*_ and *R*_*MTJ*_ of the FL of the sample for an applied magnetic field by sweeping the amplitude. The plot indicates changeable outputs caused by pinning of vortex core in different grain(s) for the applied magnetic field thus, the specific output can be obtained by choosing the amplitude accordingly.

A simplified MTJ can be thought of as comprising three key layers, namely the FL with changeable magnetization, pinned layer (PL) with fixed magnetization, and the tunneling oxide barrier. Typically, polycrystalline soft magnetic materials are used for the free layer, i.e., NiFe, FeB or CoFeB (a typical MTJ stack is shown in Fig. 1a), which will have small (i.e., 10–100 nm) crystalline grains separated by granular boundaries^31^. Different strategies can be employed to manipulate the grain structure within the NiFe layer, including (i) Changing the material of the capping layer (products adjacent to NiFe affect the grain structure during annealing), (ii) A dusting layer made of different materials (such as Ta, Ru, or W) laminated over the NiFe layer, and (iii) Annealing the MTJs at different temperatures or with different mechanisms^30,33–35^. Grains of ferromagnetic materials are inhomogeneous and approximating the behavior of different grains may result in ambiguous results^36^. The magnetic vortex core may pin in different grains with different magnetic dynamics based on the recent and previous input exhibiting different *f*_*out*_ and/or *R*_*MTJ*_ in a nonlinear manner. The abovementioned key features of a reservoir are nonlinearity and STM^12,4^, which can be found in GV-STNO devices. The pinning sites of the vortex core provide a possibility of frequency controllability of the device that can be monitored through the energy landscape of the FL^31^. Moreover, the fabricated device sample and the study of the influence of pinning sites (experimentally and micromagnetic simulations) are presented in detail by Jenkins et al.^31^. A few works have presented the pinning phenomena of vortex core due to the granular defect and how it changes the gyration frequency of the magnetic vortex depending on the pinned site^28,30,33–35^. The impact of grain boundaries on the spin wave reflection observed on spin torque oscillators is also presented in^37^. The concept of a GV-STNO-based RC system to explore the usage of grains for an electronic application is illustrated in Fig. 1 wherein (a) presents the GV-STNO nanopillar schematic, (b) is showing the concept of RC system using a GV-STNO device wherein the pinning of vortex core providing different outputs acting as reservoir neurons, and (c) demonstrates the possible nonlinearly varying outputs of the GV-STNO reservoir (*f*_*out*_ and *R*_*MTJ*_). First, the experimental measurements to attain the granular effect on both performance metrics are carried out, as presented in Fig. 2 (detailed in “Methods”). Nonlinearly varying *R*_*MTJ*_ is obtained in the range of 110 to 140 Ω for applied magnetic field with sweeping amplitude (− 14 mT to + 14 mT) on the device sample with 400 nm diameter MTJ nanopillar. Additionally, the pinning of vortex core generates nonlinearly varying *f*_*out*_ in the range of few hundred MHz up to ~ 3 GHz. By choosing a specific resistance of the vortex state and sweeping the magnetic field for a small range of the vortex core, oscillation frequency is determined through spin diode effect, as described in^22^.

## Results and discussion

### Gyrotropic frequency of magnetic vortex

**Figure 3:**
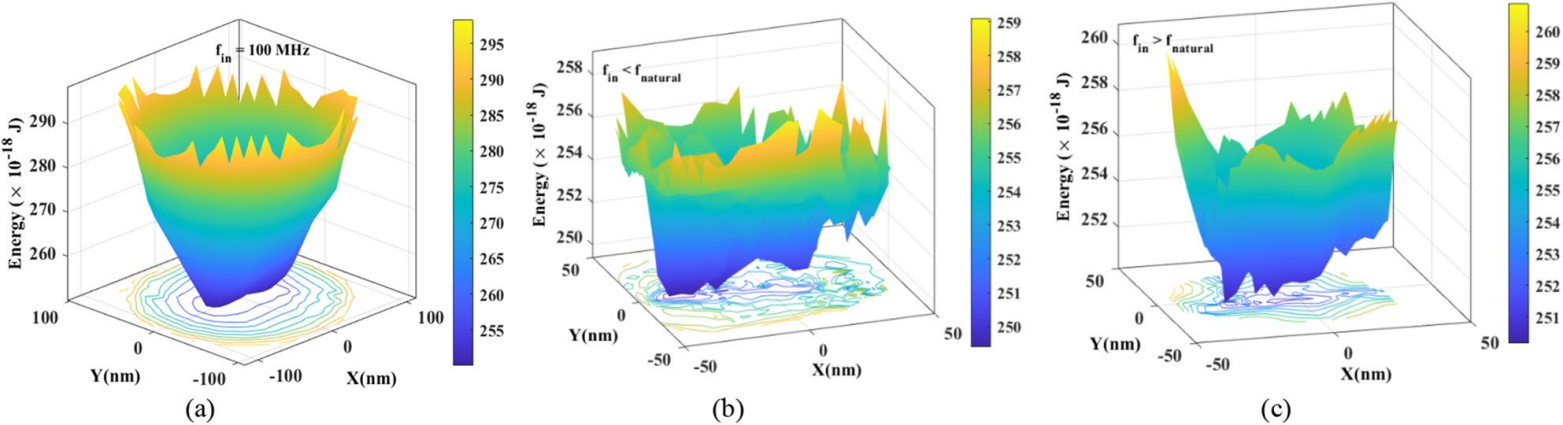
Energy landscape for three input frequencies (**a**) *f*_in_ = *f*_gyro_, (**b**) *f*_in_ < *f*_gyro,_ and (**c**) *f*_in_ > *f*_gyro_, wherein the vortex core gets pinned into the grains when input frequency is other than the natural frequency resulting in more than one energy minima (micromagnetic simulations).

Magnetic vortex core is an out-of-plane spin texture that attains a trajectory after its formation and starts oscillating due to the external applied magnetic field or DC current. The gyrotropic force, spin-transfer torque, damping, and confinement force (which mainly includes the Oersted field and magnetostatic field) governed by the Thiele equation acts on the out-of-plane magnetic vortex core while it oscillates^38,39^. The interplay between energy components, such as demagnetization energy, exchange energy, interaction energy, and Zeeman energy, determines the energy minima of the magnetic vortex. The parabolic single energy minima landscape signifies that a stable gyrotropic oscillation of the vortex core has been achieved, attaining the resonance or gyrotropic frequency (*f*_*gyro*_) of the device^31,39^. The grains present in the FL may affect the energy minimum landscape because the grains start behaving as individual magnets causing vortex core to get pinned in any of the grains. Interestingly, as per our observation, when the applied input signal is close to the *f*_*gyro*_, then the variation in energy minima is not found meaning that the energy landscape is aligned together as one energy minimum, as shown in Fig. 3a. On the other hand, the energy landscape for input frequencies of applied sinusoidal magnetic field smaller or larger than *f*_*gyro*_ shows more than one energy minima due to granularity wherein it indicates the pinning of vortex core in different grains, shown in Fig. 3b,c. Hence, when the input frequency (*f*_*in*_) close to *f*_*gyro*_, the effect of granularity is ignorable (Supplement File) and the GV-STNO behaves like an ideal MTJ without grains. Additionally, the excitation power of the vortex core should be high enough to capture the effect of granularity or oscillation. Thus, based on the data and results of this study, we propose using grains as dynamic reservoirs whereby each grain (or a few grains together) behaves as a single neuron of the reservoir producing the abovementioned different (nonlinear) *f*_*out*_ and* R*_*MTJ*_. The pinning sites which depend on the different energy barriers (with many local maxima and minima) cause the grain(s) to behave as a neuron. Each grain may act as a single neuron, however, the grains with similar properties would couple together to act as one pinning site. Hence, mapping the grains present in GV-STNO with randomly distributed reservoir neurons whose weights (different energy minima on the energy landscape or pinning sites) vary with the input and generate a nonlinear outcome. Accordingly, the FL granularity effect, which is a defect, is used as a resourceful reservoir for developing an RC system on a single device.

**Figure 4:**
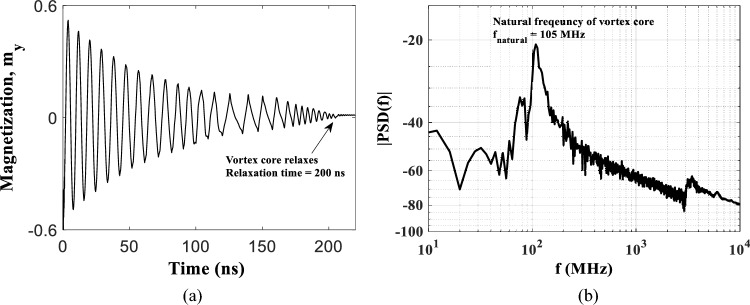
Relaxation time of 200 ns when vortex core is translated to 205 nm away (in the micromagnetic simulations) from the center which signifies the short term memory feature of RC system, and (**b**) gyrotropic frequency (~ 105 MHz) measured through FFT calculation.

We investigate *f*_*gyro*_ of the nanodisk (with diameter, D = 450 nm, FL thickness, L = 6 nm, and grain sizes 40 nm) which should remain constant with and without granular effect, for more details refer to the methods section (simulation model). The gyrotropic frequency of the device can be measured through simulation by translating or shifting the magnetic-vortex core from the center of the nanodisk by means of an initial external magnetic field, then the magnetic moment orientations in the x, y, and/or z-axis vary to attain *f*_*gyro*_ of the magnetic vortex core. The frequencies are obtained through FFT when the vortex core is translated to 205 nm away from the center. The vortex core relaxes towards the nanodisk center attaining *f*_*gyro*_ ~ 105 MHz, shown through FFT measurement in Fig. 4. The relaxation time of a magnetic vortex is the time duration that the vortex core takes to relax towards the nanodisk center from its current position in the absence of an external force (magnetic field or, DC current) and it attains its energy minima. Therefore, the closer, i.e. 10 nm (farther, i.e. 205 nm) the magnetic vortex core position, the lesser (longer) relaxation time we will see. The shortest and the longest relaxation times are measured as 35 ns and 200 ns, respectively. We run the simulation for a certain time while analyzing either the vortex core positions or the magnetization (*m*_*y*_, in this work) of GV-STNO. Moreover, 205 nm is the limit before vortex core expulsion (when the vortex core reaches the edges) takes place, thus providing the maximum relaxation time (i.e. 200 ns). Moreover, 10 nm is the nearest displacement in our case, since that vortex core did not form properly early on, possibly due to its radius. Relaxation time is acting as an STM here, if the next input is applied before the vortex core is relaxed in the center of the device, the response of the device will change depending on the location of the vortex core. In other words, the outcome depends on the previously applied signal (i.e., STM), whose amplitude or pulse width may differ. Note that the relaxation time for a device can be controlled and influenced by various factors such as geometrical size, material of FL, magnetic parameters, and so on.

Next, we studied the non-linear behavior of *f*_*out*_ and* R*_*MTJ*_, which fulfills another key feature of RC^4^. More than one non-linear parameter provides more robustness to the RC system for temporal signal analysis because it allows the acquisition of more complex and elevated associations in the input data. Considering the device's STM (or relaxation time) and non-linearity the GV-STNO appears to be a favorable option for creating a sub-micron reservoir. Henceforth, when an external magnetic field is applied to the device in the form of periodic square and/or sine waveforms, we can observe the variation in the output functionality through experimental results for 25 MHz (detailed in methods) and micromagnetic simulations for different frequencies (can be found in the next section). These output functionalities emulating reservoir neurons give rise to distinguishable characteristics enabling the classification of square and sine wave inputs. Furthermore, the variation in (1) amplitude and (2) input frequency of the applied field is carried out to study the prospects of reservoir computing using GV-STNO for waveform classification.

### Frequency bands for different functionality

Our study shows that distinct functionalities can be obtained with different input frequencies of the applied magnetic field. We divide the input frequencies of an applied magnetic field into three cases—Case I: input frequency of the applied field below *f*_*gyro*_ (*f*_*in*_ < *f*_*gyro*_), 25 and 50 MHz here, Case II: input frequency of the applied field above *f*_*gyro*_ (*f*_*in*_ > *f*_*gyro*_), 200 and 400 MHz here, and Case III: input frequencies close to *f*_*gyro*_ (*f*_*in*_ ~ *f*_*gyro*_) i.e. 105 MHz.

**Figure 5:**
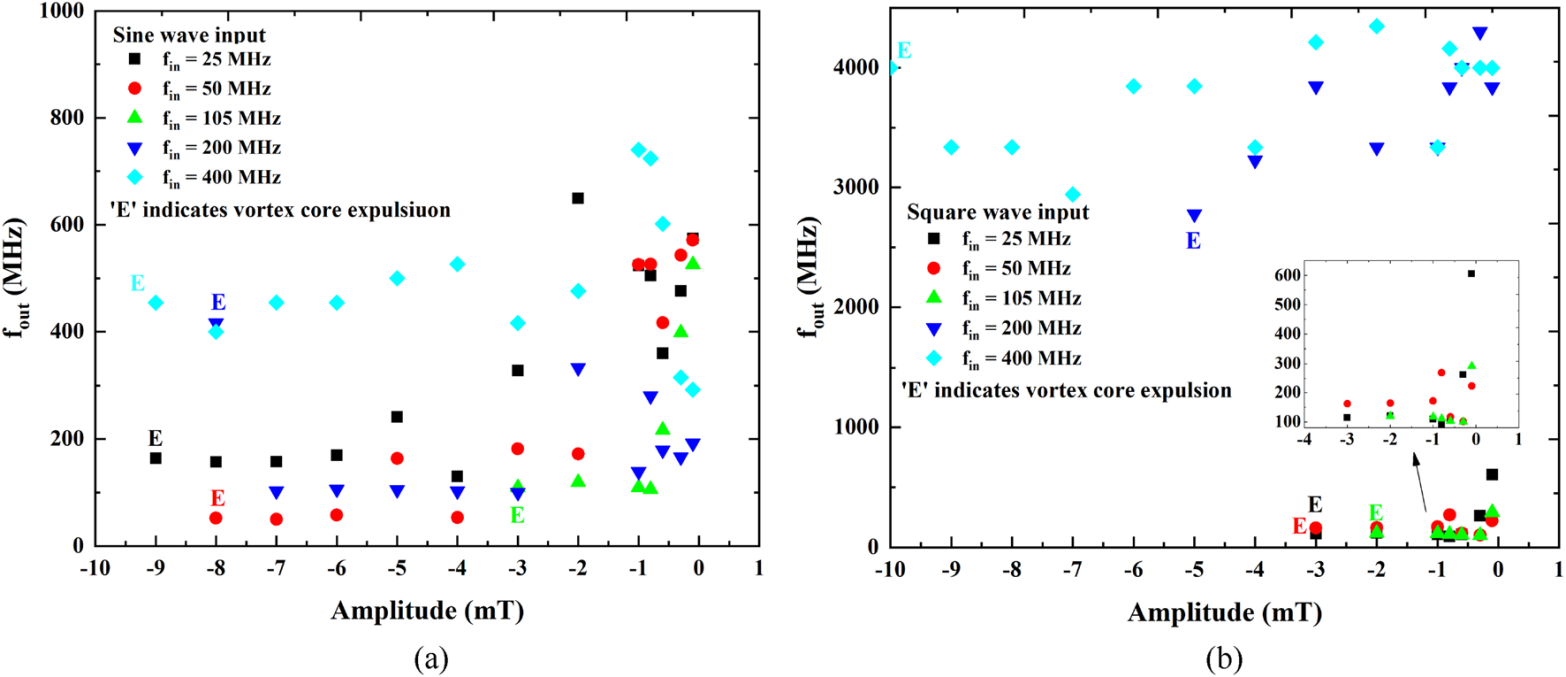
The output oscillation frequency with nonlinear characteristics (micromagnetic simulations) for (**a**) sine wave, (**b**) square wave inputs with the sweeping amplitude of the applied magnetic field with different input frequencies resulting in distinct *f*_*out*_ range. Here, E indicates the expulsion of vortex core.

**Figure 6:**
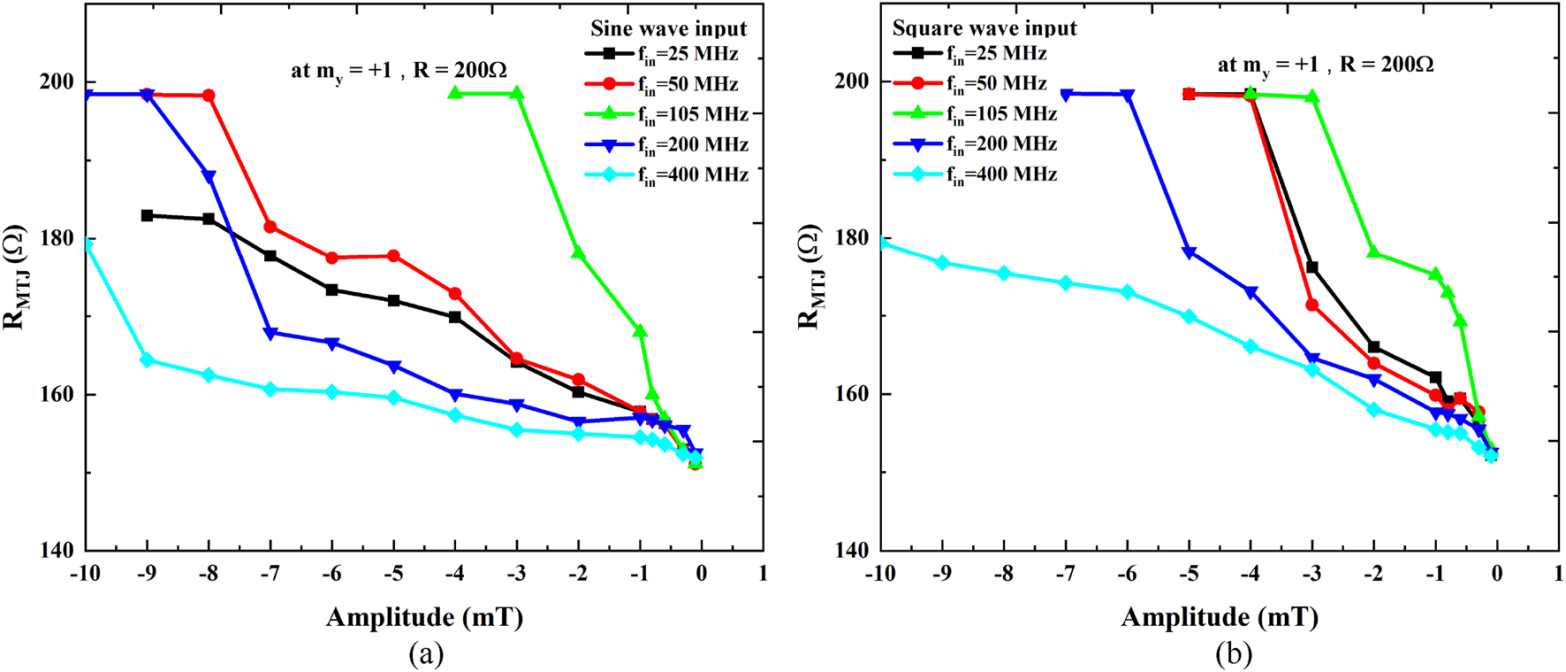
The GV-STNO nonlinear resistance in vortex state (obtained from micromagnetic simulations) for (**c**) sine wave, (**d**) square wave inputs, against the sweeping amplitude of the applied magnetic field with different input frequencies. For, *f*_*in*_ equal to *f*_*gyro*_ reaches maximum values of *R*_*MTJ*_ faster than in other cases.

A detailed study is carried out through micromagnetic simulations for different input frequencies in all three cases for said two types of input waves: sine and square wave. A thorough description of the micromagnetic simulations can be found in the method section. In this study, the amplitude of the applied magnetic field is swept (0.1 mT to 10 mT) for three cases to measure the *f*_*out*_ (Fig. 5a,b) and *R*_*MTJ*_ (Fig. 6a,b). As shown in Fig. 5a,b, *f*_*out*_ is changing nonlinearly by increasing the amplitude for both sine and square waveforms, respectively. This is because the oscillation frequency of various grains can differ significantly, and adjusting the amplitude of the input field can cause the vortex core to be pinned in different grains, like observed experimentally (Fig. 2). One of the most interesting features of the GV-STNOs is the highly nonlinear change of *f*_*out*_ that makes them a promising candidate as a neuron in RC systems. Another interesting feature of the GV-STNO devices is that for *f*_*in*_ close to the *f*_*gyro*_ (e.g., *f*_*in*_ = 105 MHz in Fig. 5a,b), the vortex core tends to stop pining in the grains with relatively higher input amplitude (> 1mT) irrespective of the input waveform sine or square. This also satisfies the reasoning behind the nullification of the granular effect for Case III. For case I, both sine and square inputs generate *f*_*out*_ ranging from 50 to 600 MHz, however, the expulsion of vortex core happens earlier for square waves than for sine. The early expulsion for square wave inputs is probably due to having the maximum amplitude throughout the time period as compared to sine which is incremental in nature. Lastly, for case II, the *f*_*out*_ for sine inputs almost remains closer to *f*_*in*_ while for square waves higher *f*_*out*_ (ranging from 2.5 to 4.5 GHz) is obtained. Moreover, as we keep increasing the amplitude, the vortex core will expel, denoted as E for each input frequency (more details in Supplement File). Again, for gyrotropic frequency (case III), due to its higher output power, the expulsion occurs faster than other cases.

In this study, we also measured *R*_*MTJ*_ versus the amplitude of the input magnetic field for both waveforms. *R*_*MTJ*_ is extracted from *m*_*y*_ considering approximation as, *R*_*MTJ*_ = ((*m*_*y*_ × *MR*) + *R*_*DC*_) where MR = 100% (experimentally it might be a variable parameter) and *R*_*DC*_ = 150 Ω. The resistance plots in Fig. 6a,b for sine and square wave inputs, respectively, reveal that changes in resistance are also non-linear with respect to the input signal amplitude. Moreover, these characteristics are similar to the experimental variation shown in Fig. 2. However, the degree of non-linearity in *R*_*MTJ*_ changes is significantly lower than that observed for *f*_*out*_. The resistance increases as we increase the amplitude of the applied field with sine wave inputs, however, at 400 MHz, the increase rate is slower (light blue curve in Fig. 6). On the other hand, for square wave inputs shown in Fig. 6b, the resistance increase rate with respect to amplitude is faster for case I when compared to Case II. It is worth noting that for Case III, *f*_*in*_ = 105 MHz, resistance reaches its maximum value for lower input signal amplitude irrespective of the input waveform sine or square, as expected. Hence, depending on the intended application of an RC system, either *f*_*out*_, R_MTJ_, or both can be utilized for classification purposes. Both sine and square wave input signals show distinct responses toward the amplitude sweep and different input frequencies.

### Sine and square wave classification

**Figure 7:**
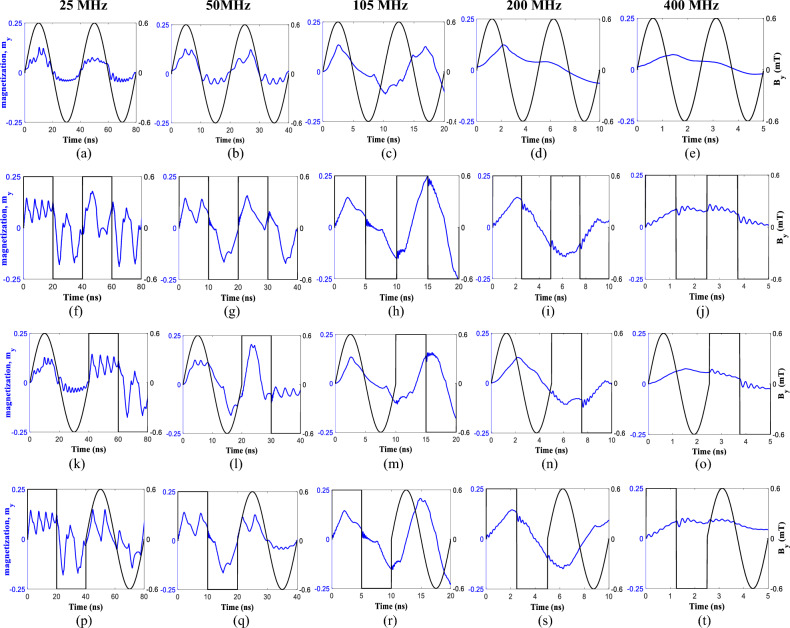
Magnetization and output frequency response of the magnetic vortex core due to the granular pinning for a combination of sine and square wave inputs as training sets during micromagnetic simulations, (**a**–**e**) sine-sine, (**f**–**j**) square-square, (**k**–**o**) sine-square, and (**p**–**t**) square-sine with 0.6 mT amplitude and different input frequencies of 25, 50, 105, 200, and 400 MHz. the output waveforms in blue are used to calculate the *f*_*out*_ while *m*_*y*_ is used to evaluate the *R*_*MTJ*_. For Case I, higher *f*_*out*_ for sine waves while higher *m*_*y*_ for square waves are observed. On contrast, for Case III, higher *f*_*out*_ for square waves are speculated. Moreover, for combinational waves (**k**–**t**), the STM effects is observed. FFT of the sine and square wave inputs (for given frequencies) are presented in the Supplement File.

Next, for waveform classification as a demonstration of a simple temporal pattern analysis, we studied the response of the device performance for four combinations of the signals including sine-sine (Fig. 7a–e), square-square (Fig. 7f–j), sine-square (Fig. 7k–o), and finally square-sine (Fig. 7p–t) as a training set for the RC system, each with the five input frequencies (25, 50, 105, 200, and 400 MHz). The training sets for said input frequencies and reservoir output characteristics are illustrated in Fig. 7. Worth noting, the major advantage of the presence of grains on the figure-of-merit of the system are as follows: (1) due to the pinning effect, a single device generates more than one (and nonlinear) *f*_*out*_ peaks which in case of without grains at most two *f*_*out*_ are probable (at *f*_*in*_ and *f*_*gyro*_) and (2) higher output power is achieved in a system with grains making it easier to detect the signal by the readout circuits (more information in Supplement File).The readout layer can be trained based on these training sets consisting nonlinearity and STM which later helps to classify the input waveform.

**Figure 8:**
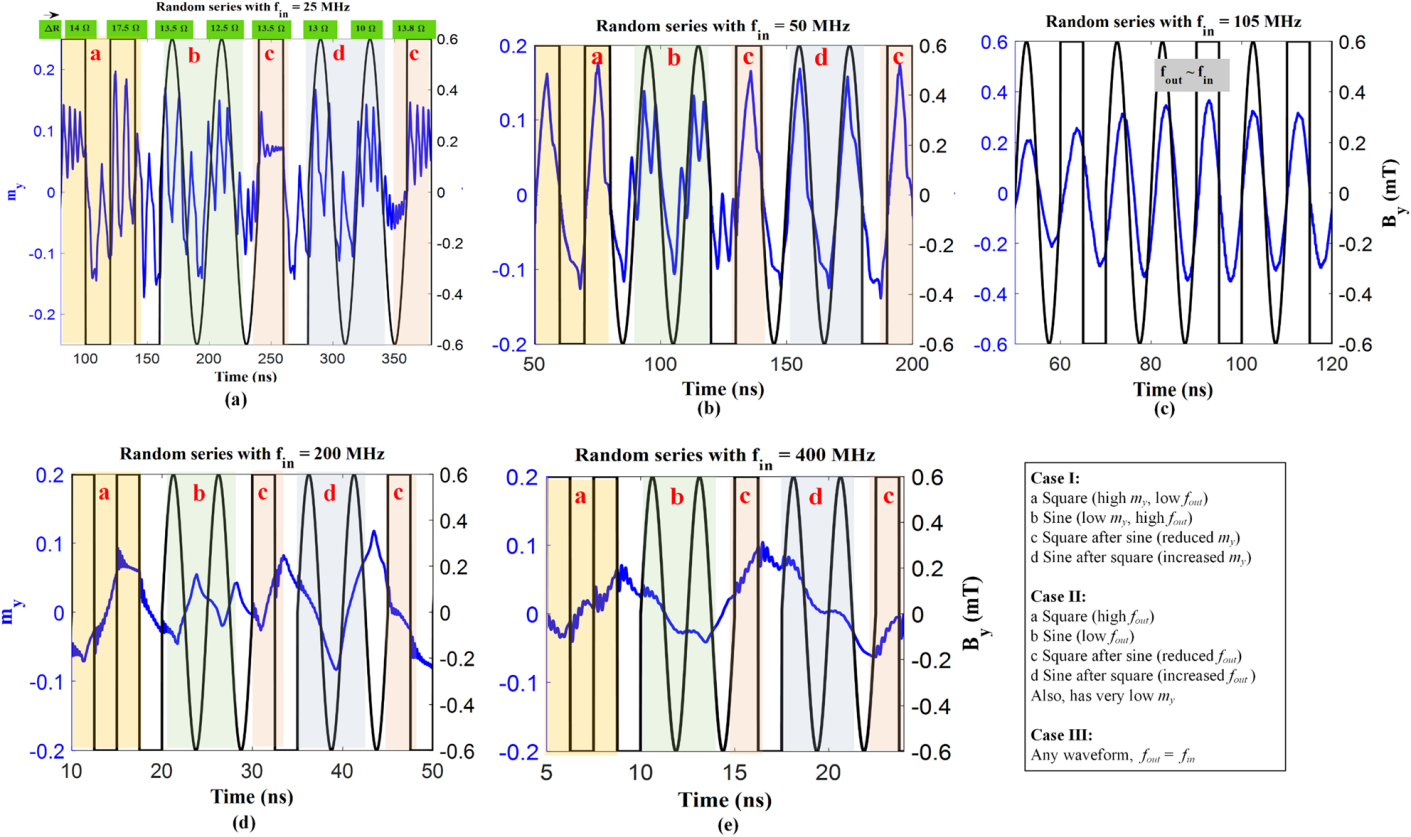
The output magnetization (*m*_*y*_) for a given random sine-square input waveforms (with magnetic field (*B*_*y*_) as 0.6 mT) obtained from micromagnetic simulations with varying input frequencies (**a**) 25 MHz, (**b**) 50 MHz, (**c**) 105 MHz, (**d**) 200 MHz, and (**e**) 400 MHz, the response of *m*_*y*_ and oscillation frequency (*f*_*osc*_) plays the role of the output of reservoir computing (Cases described in frequency bands for different functionality section). Colored bars are labeled showing different output characteristics which are mentioned inside the box with three cases.

Henceforth, when random sine and square wave input signals as test set (20 time periods, selected as shown in Fig. 8) are applied then the classification will be carried out in the aforesaid three cases. For case I, depending on the increasing resistance when compared to the preceding signal, the classification can be performed. For instance, for each increasing ΔR, the signal is square, while a decreasing value signifies it to be a sine wave as shown in Fig. 8a. However, for square-sine wave input, the increment in *m*_*y*_ (also, ΔR) is clear from Fig. 8a,b. This justifies the STM feature of the RC as well wherein the preceding input has an impact on the proceeding signal. Next, for case II, we see higher *f*_*out*_ for square wave signals which is not the case for sine waves (Refer to Fig. 8d,e). Additionally, for these frequencies, the value of *m*_*y*_ is very low to readout, thus, *f*_*out*_ can be used to distinguish the waveforms. Furthermore, for case III, *f*_*out*_ becomes the same as *f*_*gyro*_ nullifying the granular effects after a couple of cycles, as indicated in Fig. 8c. In waveform classification, RC excels because it can handle nonlinear and time-varying inputs that are difficult to model using conventional machine learning methods. Henceforth, the waveform classification concept using GV-STNO can be applied to multiple applications with inputs such as recorded audio signals, electrocardiograms (ECGs) or electroencephalograms (EEGs), seismic signals, temporal signals, and so on. Moreover, to reduce the complexity of having different classification approaches for case I and II which, we observed in our investigation, we can control the gyrotropic frequency according to the requirement of application by means of variation in the aspect ratio of nanopillar.

## Conclusions

In this work, a magnetic tunnel junction has been experimentally demonstrated to exhibit nonlinear characteristics due to grains in the free layer which affect the resistance of the vortex state as well as the output oscillation frequency. The vortex core pinned in different grains is shown to produce a wide range of frequencies from a few hundred MHz up to 5 GHz within a single device. Owing to the short-term memory capacity in the form of relaxation time of the vortex core along with the nonlinear dynamics in terms of resistance and output oscillation frequency, a reservoir computing system implemented with only one single GV-STNO is presented. In particular, we investigated the waveform classification task using the different pinning sites caused by the grains acting as reservoir neurons. Furthermore, sine and square waveforms with varying pulse width and amplitude were applied to the system for training whereas random sine-square signals were examined through reservoir features of non-linearity and short term memory for classification purpose. Therefore, GV-STNOs could be a promising candidate for future unconventional computing paradigms due to their randomness and their ability to change resistance and output frequency nonlinearly as a result of the granular pinning of the magnetic vortex core. Consequently, one spintronics nanodevice, wherein multiple grains function as neurons and their nonlinear weights as synapses can be used for a wide range of applications through reservoir computing. Hence, GV-STNO has potential to unlock several other technological advancements towards spaciotemporal and similar applications.

## Methods

### Simulation model

MuMax3 tool is used to perform all the micromagnetic simulations^40^. The proof of concept for reservoir computing for waveform classification is carried out using micromagnetic simulations after studying and measuring the granularity effect in the device sample. The device parameters considered for NiFe are: diameter, *D* = 450 nm, L = 6 nm, exchange stiffness, *A*_*ex*_ = 1.3 × 10^–11^ J, saturation magnetization, *M*_*s*_ = 740 kA/m, damping coefficient, *α* = 0.01, polarization, *P* = 0.5, and exchange length, *L*_*ex*_ = 6.15 nm. The grain size considered here is 40 nm and random variation of 10% is applied to saturation magnetization which generates random pinning sites. Moreover, exchange coupling between grains is reduced to 0.9*A*_*ex*_ at the grain interfaces^31^. Furthermore, to investigate the behavior of granularity for different types of input magnetic field (in-plane) waves, the sine and square wave inputs are applied to the system. The amplitude and pulse width of the input signal is varied to monitor the non-linearity and short-term memory key features necessary for reservoir computing. The pulse widths used for applied sine and square magnetic field signals are 40, 20, 10, 5, and 2.5 ns and the simulation run time is kept for two time periods while saving the outputs (such as magnetization, core position, energy, and so on) every 10 ps. The initial relaxation time of the vortex core is not incorporated in the result section to preserve the dynamic component only, moreover, by subtracting relaxation time we are able to calculate the frequency through FFT more effectively. Furthermore, the amplitude of the applied magnetic field varies from 1 to 10 mT (or, until vortex expulsion took place). Later, the random signal (combination of sine and square pulses) is applied for classification purposes. The built-in vortex initialization syntax of MuMax3 is used to obtain the vortex state. Magnetization, *m*_*y*_, is the main resource to calculate the MTJ resistance, *R*_*MTJ*_ which can further be used for calculation of the oscillation output power for readout. Moreover, power spectral density is evaluated to measure the granular output frequency wherein the maximum frequency for specific applied input is reported in this work.

### Measurement process

**Figure 9:**
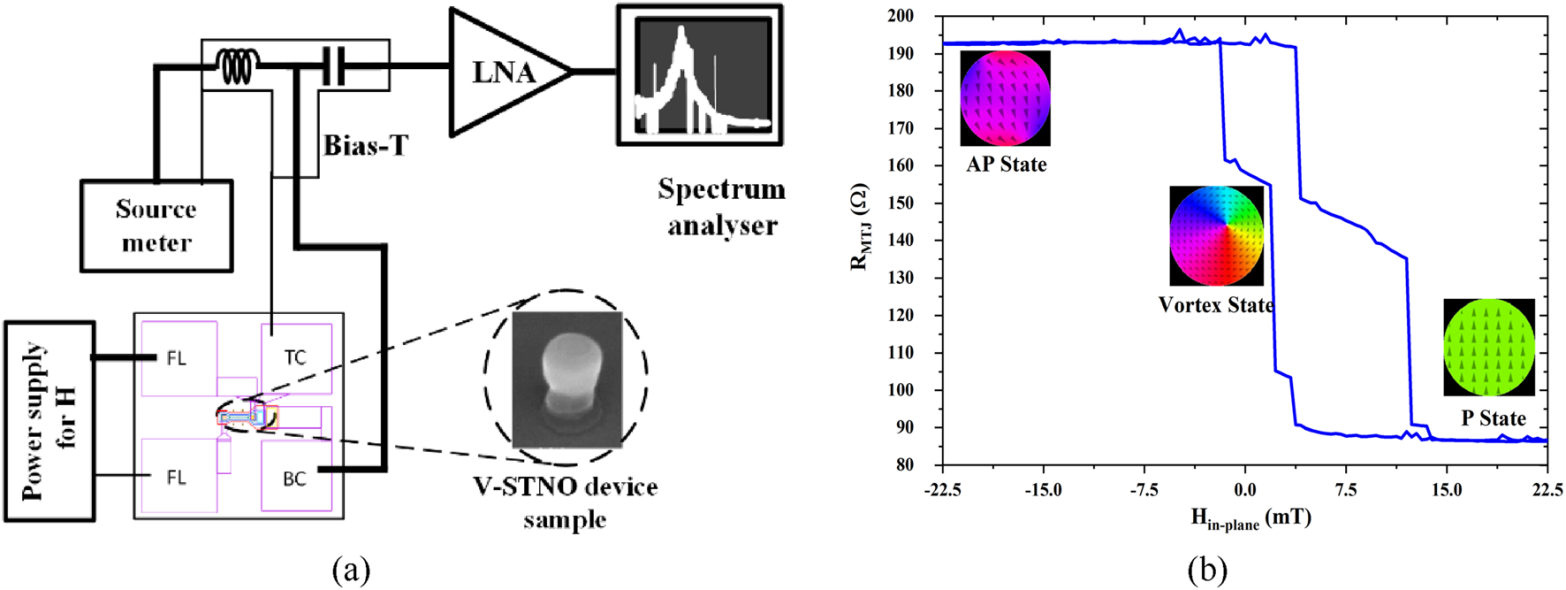
(**a**) The sample measurement setup with the device sample connected to the power supply, source meter, and bias tee while frequency is measured using a spectrum analyzer, and (**b**) experimental measurement of transfer curve of device sample at low bias current to obtain resistance states.

**Figure 10:**
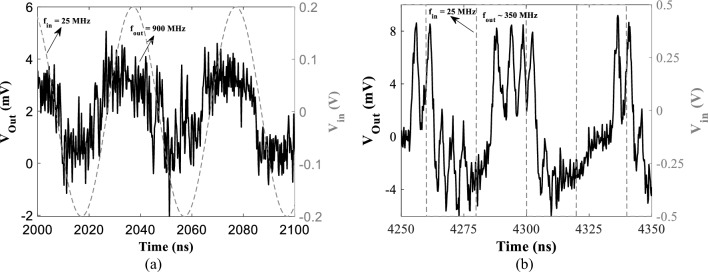
Experimental measurement of the device sample for (**a**) sine and (**b**) square input waveforms with *f*_*in*_ = 25 MHz with a response showing output pinned oscillation frequency of approximately 900 MHz and 350 MHz for sine and square inputs, respectively.

The measurement setup is presented in Fig. 9a, the measurement is only performed for the device sample for the granularity effect. The MTJ sample of 400 nm diameter is used in this work is comprised of /5 Ru/6 IrMn/2.0 CoFe30/0.7 Ru/2.6 CoFe_40_B_20_/1.0 MgO/2.0 CoFe_40_B_20_/0.2 Ta/7.0 NiFe/10 Ta/7.0 Ru (thicknesses in nm), where the 2.6 CoFe_40_B_20_ is the top layer of the synthetic anti-ferromagnet acting as the polariser layer and the composite 2.0 CoFe_40_B_20_/0.2 Ta/5.0 NiFe layer is the FL and is assumed to be sufficiently coupled as to act as a single magnetic layer (refer to Fig. 1)^41^. The measurement starts by applying a low reading current (50 µA) to the STNO through an RF probe with ground and signal tip to obtain the P and AP resistance, thus, the TMR, refer to Fig. 9b. The transfer curve of the device which contains the resistance of MTJ (*R*_*MTJ*_) versus the applied in-plane field is measured by sweeping the current of the external electromagnet while reading *R*_*MTJ*_ using source meters. The magnetic field strength (H_in-plane_) of the electromagnet is converted from its current (I_in-plane_) by calibration using a Tesla meter. I_in-plane_ starts with − 200 mA with a 5 mA positive step size until it reaches 200 mA, then decreases with a 5 mA step until it returns to − 200 mA. Afterward, to obtain the vortex state, a higher read current (> I_th,vortex_) is used passing through the MTJ while sweeping the magnet field to get the device into vortex core oscillation mode. The vortex states are generally noticed along the process when MTJ changes to AP state from P state. The nonlinearity present in the vortex resistance is due to the granular effect present in the free layer. The oscillation is found using a setup that contains a bias tee and a spectrum analyzer in addition to the transfer curve measurement, sweeping the magnetic field within the vortex range with small steps while applying a bias current to the MTJ and monitoring the spectrum analyzer. Furthermore, experimental measurements for sine and square waveforms with *f*_*in*_ = 25 MHz are performed wherein *f*_*out*_ as approximately 900 MHz and 350 MHz, respectively, are attained (refer to Fig. 10a,b) which align with the simulation results. Here, an AC voltage is applied to an integrated field line located 400 nm above the MTJ, which results in an in-plane magnetic field equivalent to roughly 0.1mT/mA at the MTJ. The AC voltage across the MTJ is measured via the RF channel of a bias tee and is presented as a function of time, for an excitation signal of 25 MHz and *V*_*in*_ as shown in Fig. 10 applied to the field line, for both sine and square waves.

### Supplementary Information

Supplementary Information.

## Data Availability

The data that support the plots within this paper and other findings of this study are available from the corresponding author at a reasonable request.
