# Supplementary material for: Granular vortex spin-torque nano oscillator for reservoir computing
Source: Sci Rep. 2023 Oct 4;13:16722. doi: 10.1038/s41598-023-43923-z (PMC10550924; doi:10.1038/s41598-023-43923-z)
Supplement: Supplementary file 1 — Supplementary Information. [file 41598_2023_43923_MOESM1_ESM.pdf]

Granular Vortex Spin-Torque Nano Oscillator for Reservoir Computing

S. Shreya[1], A. S. Jenkins[2], Y. Rezaeiyan[1], R. Li[1], T. Böhnert[2], L. Benetti[2], R. Ferreira[2], F. Moradi[1], and H. Farkhani[1]
[1] Electrical and Computer Engineering Department, Aarhus University, Aarhus 8200, Denmark
[2] International Iberian Nanotechnology Laboratory (INL), Braga, Portugal

Supplement Information

The grains present in the free layer of the magnetic tunnel junction and their effect can be easily monitored through the energy landscape. In the main text, Fig. 2 shows how different input frequencies (equal, less, and greater than natural frequency) change the energy landscape due to the interplay between the several energy components exchange, demagnetization, and Zeeman. Fig. S1 shows that there is one local energy minimum for the natural frequency of the magnetic vortex core, f_{gyro} irrespective of the presence or absence of grains [1]. The vortex core is translated to 100 nm distance from the center.

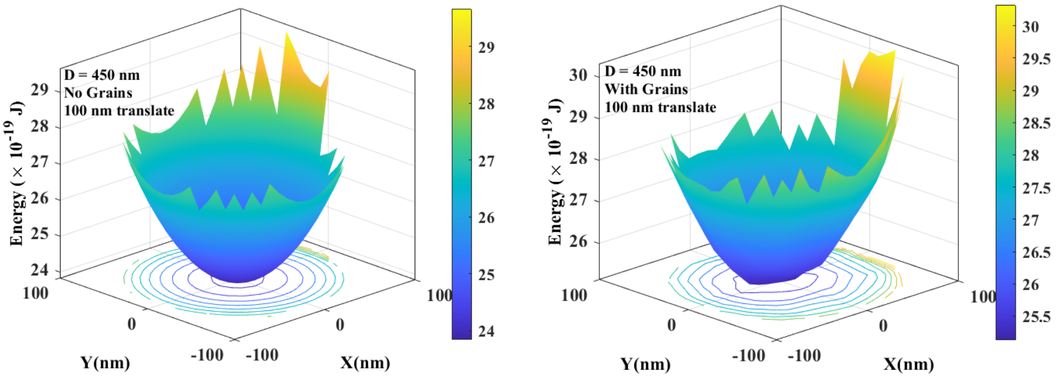

Fig. S1. Total energy density landscape of GV-STNO plotted against the circular replacement of the vortex core position on the FL surface considering (a) no granular effect and (b) with granular effect.

The nonlinearity observed for the MTJ resistance experimentally (refer to Fig. 3 (c)) indicates the presence of a granular effect in the system otherwise the relation is supposed to be linear [2]. Moreover, the randomness in the nonlinear characteristics of the resistance in the vortex state for different devices with varying diameters is also observed and presented in Fig. S2. Consequently, the experiment provides proof of the concept that reservoir computing can be a good candidate for future applications provided by granular effects present in the system.

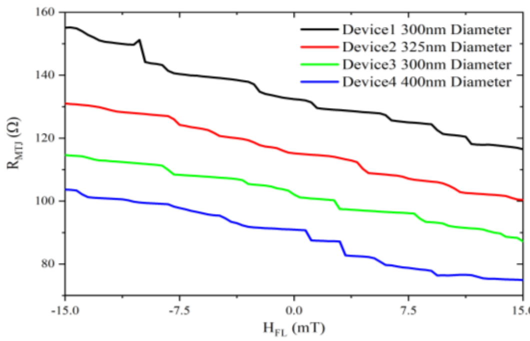

Fig. S2. For different samples, the MTJ resistance in the vortex state shows randomness when it comes to the nonlinear behavior caused by the grains.

Fig. 4 of the main text wherein the amplitude of the applied Magnetic field is varied to investigate the nonlinear feature of reservoir computing. The output frequency and MTJ resistance for different amplitudes for sine and square wave inputs are presented here in Table 1. Moreover, the MTJ resistance is calculated as $R_{MTJ} = ((m_y \times MR) + R_{DC})$ for which the maximum value of m_y is considered. This performance metric is useful for reading out through a CMOS circuitry.

Table 1: Oscillation frequency and MTJ resistance for sine and square wave inputs with different amplitude and input frequencies

f_{in}		25 MHz		50 MHz		105 MHz		200 MHz		400 MHz	
Output parameters/ Input amplitude (mT)	Input signal	f_{out} (MHz)	R_{MTJ}(Ω)	f_{out} (MHz)	R_{MTJ} (Ω)	f_{out} (MHz)	R_{MTJ} (Ω)	f_{out} (MHz)	R_{MTJ} (Ω)	f_{out} (MHz)	R_{MTJ} (Ω)
0.1	Sine	574.7	151.8	571.4	151.15	526	151.15	192	152.5	292	151.95
	Square	606.1	152.2	222.2	152.45	291.5	152.95	3840	152.5	4000	152.1
0.3	Sine	476.2	153	543.4	152.9	398.4	152.9	166	155.5	315	152.4
	Square	263.1	156.2	101.5	157.7	100	157.15	4310	155.5	4000	153.2
0.6	Sine	359.7	156.3	416.6	156.2	217.4	156.9	179	156.1	602	153.6
	Square	108.7	159.1	117	159.4	103.1	169.25	4000	156.8	4000	155.1
1	Sine	523.5	157.8	525.5	157.8	109.8	168	138.8	157.1	740	154.5
	Square	109.8	162.1	171.2	159.8	117.6	175.2	3333	157.5	3333	155.5
3	Sine	327.8	164.15	181.81	164.6	109.8	198.5	100.2	158.8	416.6	155.5
	Square	114.94	176.2	162.1	171.4	-	198.5	3846	164.9	4214	163.1
5	Sine	169.5	173.4	58	177.5	-	-	106.4	164.6	454.5	160.4
	Square	-	198.4	-	198.4	-	-	-	174.6	3846	169.9
8	Sine	157.2	182.5	52	198.3	-	-	416.6	188.1	400.1	162.5
	Square	-	-	-	-	-	-	-	198.4	3333	175.4

Table 2: Oscillation frequency and AC component of resistance for different combinations of sine and square wave inputs with different input frequencies

f_{in}	25 MHz		50 MHz		100 MHz		200 MHz		400 MHz	
Input signal/parameters	f_{osc} (MHz)	ΔR (Ω)	f_{osc} (MHz)	ΔR (Ω)	f_{osc} (MHz)	ΔR (Ω)	f_{osc} (MHz)	ΔR (Ω)	f_{osc} (MHz)	ΔR(Ω)
Sine-Sine	400-500	16.5	300-450	9.5	80-100	11	250	9.5	660	5
Square-Square	120-200	19	140-180	16.5	85-100	25	300-350	14	250-300	5
Sine-Square	180-238	15.5	175-500	15	83-110	21	Up to 1000	14	Up to 4000	5
Square-Sine	166-450	16.2	200-350	17.85	78-120	12.85	Up to 1000	12.5	Up to 4000	4.5

For the waveform classification application, we have considered four different combinations of sine and square waveform inputs– Sine-Sine, Sine-Square, Square-Sine, and Square-Square as presented in Fig. 5 of the main text. Table 2 presents the frequency range of pinned vortex core (f_{out}) and oscillating resistance (ΔR) (which gives rise to MTJ resistance) for four combinational signals.

For Fig. 7 in the main text, we present the frequency detection through the spin diode effect. A front-end CMOS circuit is also proposed [2] that generates a DC voltage if and only if the frequency range of the input signal is closed to the oscillation frequency of the nano-oscillator, which mimics a down-converter mixer followed by a low-pass filter with an ultra-high-quality factor or an ideal in-phase only locking amplifier. This suppresses all out-of-band noise and allows the output signal to be detected. To prove this claim, a noisy and pure sinusoidal signal has been applied to the system. Similarly, if we want to detect the granular frequency, then we might need a filter to choose the specific band of granular output frequency while discarding the input frequency. For instance, we performed FFT for input sine and square wave signals with frequencies 25 and 50 MHz, respectively. As shown in Figs. S3 and S4, if input frequency (with high power though) will be filtered, then on amplifying the power of granular frequency we can detect the grain frequency.

FFT signals and their description for sine and square wave inputs for without (wo) and with (w) grain systems are shown hereby:

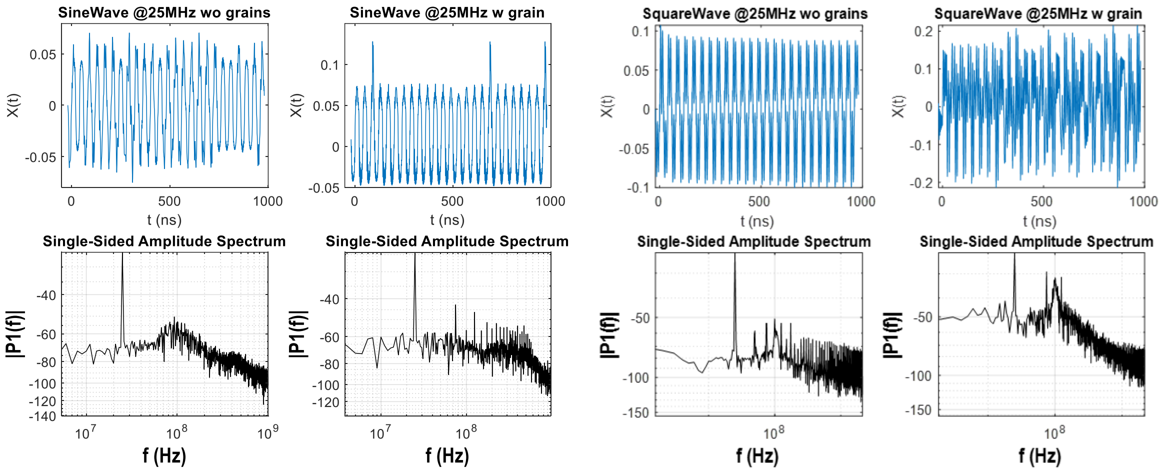

Fig. S3: FFT of output signal for given input frequency as 25 MHz for sine and square waveforms wherein the with grain system shows more than one output power peak due to f_{out} obtained at different pinning sites

Sine input: The power at input frequency (25 MHz), without grain is -25.8 dBm whereas for with grains -24.2 dBm. Moreover, there is no other peak frequency for without grains system (maybe a slight peak at gyrotropic frequency) whereas we can see some peaks for with grains system (at 75 MHz, 125 MHz, 225 MHz, and 473 MHz with power -42 dBm, -46.2 dBm, -52 dBm, and -53 dBm, respectively).

Square input: The power at input frequency (25MHz), without grain is -23.68 dBm whereas for with grains it is -23.3 dBm. Moreover, there is no other peak frequency for the without grains system (except at gyrotropic frequency) whereas we can see some peaks for with grains system (at 74.8 MHz, 100 MHz, and 124 MHz with power -29.3 dBm, -31.9 dBm, and -31.2 dBm, respectively). Moreover, for the square wave input, we see some harmonics in the without grains system too but those signals are too weak which could be of the order of thermal noise floor.

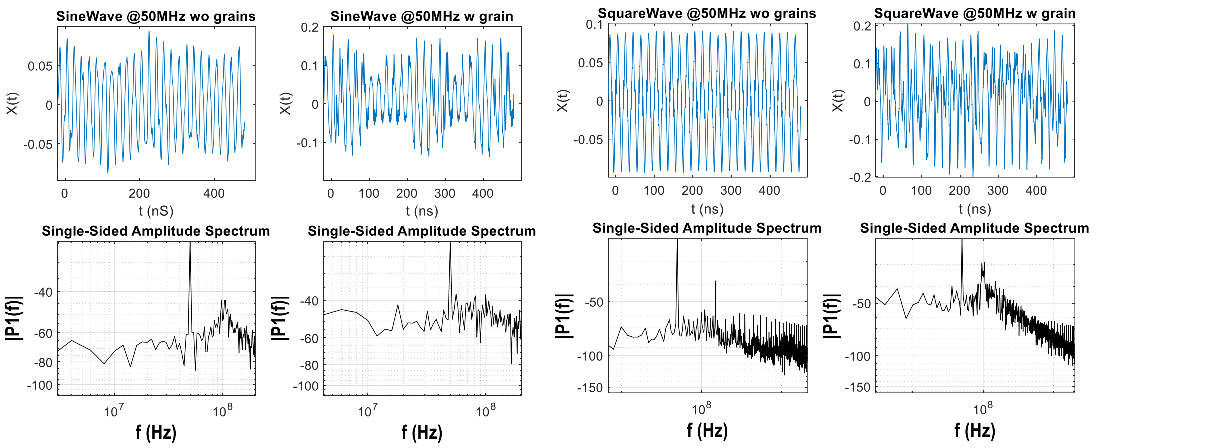

Fig. S4: FFT of output signal for given input frequency as 50 MHz for sine and square waveforms wherein the with grain system shows more than one output power peak due to f_{out} obtained at different pinning sites

Sine input: The power at input frequency (50MHz), without grain is -24.4 dBm whereas for with grains -21.2 dBm. Moreover, there is no other peak frequency for the without grains system (except at gyrotropic frequency) whereas we can see some peaks for with grains system (at 55 MHz, 81.8 MHz, and 100 MHz with power -37.3 dBm, -38.1 dBm, and -37.6 dBm, respectively).

Square input: The power at input frequency (50MHz), without grain is -22.2 dBm whereas for with grains -21.3 dBm. Moreover, there is no other peak frequency for the without grains system (except at gyrotropic frequency) whereas we can see some peaks for with grains system (at 83.8 MHz, 95 MHz, 104 MHz, and 149 MHz with power -38.7, -29.6, -29.2, and -37.6 dBm, respectively).

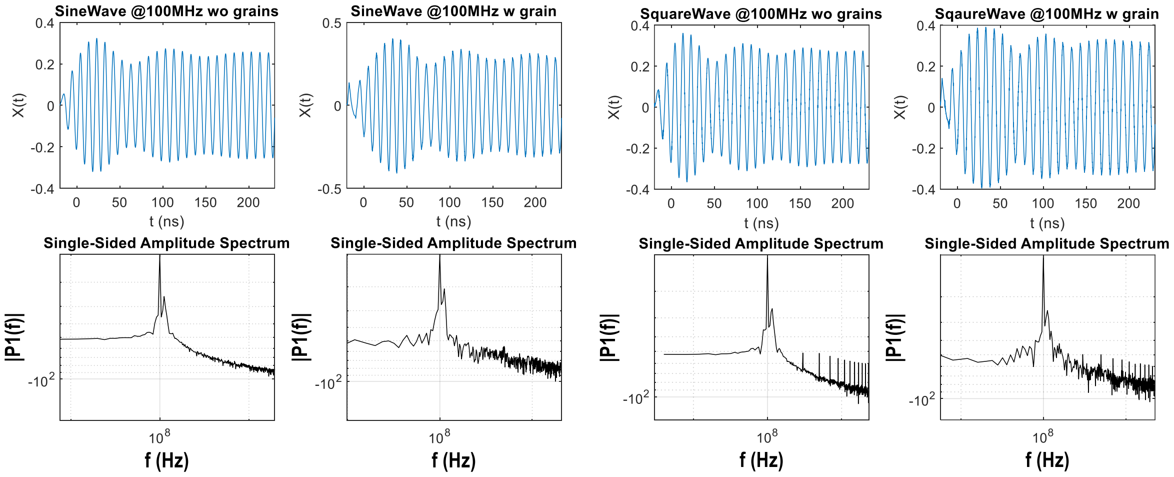

Fig. S5: FFT of output signal for given input frequency as 100 MHz for sine and square waveforms wherein the without and with grain system only show one high output peak power at the input frequency which is the gyrotropic frequency.

Sine input: The power for resonant frequency (at 100 MHz) is -12.5 dBm and -11.4 dBm, for without grains and with grains systems, respectively.

Square input: The power for resonant frequency (at 100 MHz) is -11.5 dBm and -10.3 dBm, for without grains and with grains systems, respectively.

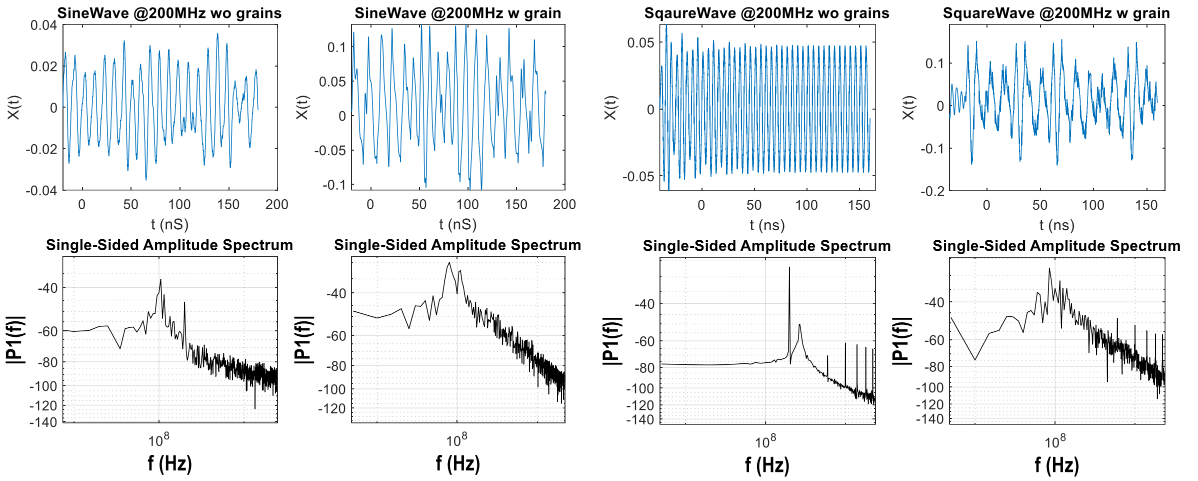

Fig. S6: FFT of output signal for given input frequency as 200 MHz for sine and square waveforms wherein the with grain system shows more than one output power peak due to f_{out} obtained at different pinning sites

Sine input: The power at input frequency (200 MHz), without grain is -45.8 dBm whereas for with grains there is no frequency peak at input signal. Moreover, there is no other peak frequency for the without grains system (except at gyrotropic frequency) whereas we can see some peaks for with grains system (at 50 MHz, 80 MHz, and 109 MHz with power -41.1 dBm, -29.8 dBm, and -32.3 dBm, respectively).

Square input: The power at input frequency (200 MHz), without grain is –26.9 dBm whereas for with grains there is no frequency peak at input signal. Moreover, there is no other peak frequency for the without grains system (except at gyrotropic frequency) whereas we can see some peaks for with grains system (at 59.7 MHz, 84.7 MHz, 100 MHz, 115 MHz, 125 MHz, 139.5 MHz, 598 MHz, and 1 GHz with power -36 dBm, -27.7 dBm, -34.6 dBm, -31.8 dBm, -34.4 dBm, -34.6 dBm, -47.6 dBm, and -51 dBm, respectively). Interestingly, we also see some high-frequency peaks in the without grains system, however, the signals are too weak to be detected by CMOS readout circuit whereas the system with grains provides more f_{out} peaks with detectable output power.

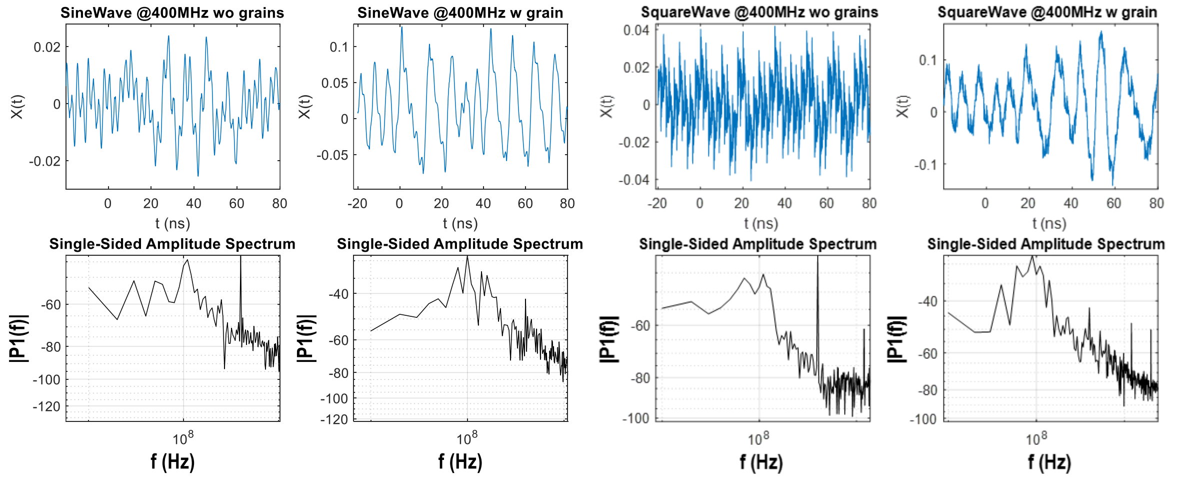

Fig. S7: FFT of output signal for given input frequency as 400 MHz for sine and square waveforms wherein the with grain system shows more than one output power peak due to f_{out} obtained at different pinning sites

Sine input: The power at input frequency (400 MHz), without grain is -43 dBm whereas for with grains it is -41.9 dBm. Moreover, there is no other peak frequency for the without grains system (except at gyrotropic frequency) whereas we can see some peaks for with grains system (at 80 MHz, 100 MHz, 140 MHz, 160 MHz, and 219.5 MHz with power -31.8 dBm, -28.6 dBm, -33 dBm, -34.1 dBm, and -43.6 dBm, respectively).

Square input: The power at input frequency (400 MHz), without grain is -43 dBm whereas for with grains it is -41.9 dBm. Moreover, there is no other peak frequency for the without grains system (except at gyrotropic frequency) whereas we can see some peaks for with grains system (at 40 MHz, 60 MHz, 90 MHz, 139.7 MHz,

169.6 MHz, 219 MHz, 400 MHz, 528.8 MHz, 1.2 GHz, and 2 GHz with power -35.2 dBm, -30.4 dBm, -28 dBm, -34 dBm, -44 dBm, -45.9 dBm, -42.2 dBm, -50.3 dBm, -47.6 dBm, and -50 dBm respectively). We can also see some high-frequency peaks in the without grain system which might be due to the harmonics.

The oscillation power can be evaluated using the following expression [4]:

$$P_{out} = \frac{(I_{read})^2}{4} \frac{(\Delta R)^2 . R_L}{(R_L + R_{DC})^2} \tag{1}$$

where ΔR is the AC resistance which is the product of m_y with magnetoresistance (MR) and R_{DC} is the average of P and AP resistances (here, 100 Ω and 200 Ω, respectively). Further, the load resistance, R_L, is 50 Ω which is also used for impedance matching. I_{read} is the applied current to read out the vortex state of the nanodisk. The readability in some cases is low, especially for lower amplitude of the applied fields, however, it is much higher when compared to skyrmions [5]. The magnetization, m_y, for skyrmions, is predicted to be less than 0.1 through the simulation as reported in [5] and [6], however, using GV-STNO, a higher and easily detectable outcome is achieved.

References:

1. S. Shreya, A. Jenkins, T. Böhnert, R. Ferreira, F. Moradi and H. Farkhani, "Frequency Sensing and Detection using Granular Vortex MTJ Nano Oscillator," *2023 IEEE International Magnetic Conference - Short Papers (INTERMAG Short Papers)*, Sendai, Japan, 2023, pp. 1-2, doi: 10.1109/INTERMAGShortPapers58606.2023.10228828.
2. Jenkins, A.S., et al., *Spin-torque resonant expulsion of the vortex core for an efficient radiofrequency detection scheme.* Nat Nanotechnol, 2016. 11(4): p. 360-4.
3. Y. Rezaeiyan, M. Zamani, S. Shreya, H. Farkhani, and F. Moradi, "Spin-Torque Based Radio-Frequency Signal Classification Front-End", IEEE ISCAS 21-25th May 2023, Monterey, CA, USA.
4. Shreya, S., et al., *Memory and Communication-in-Logic Using Vortex and Precessional Oscillations in a Magnetic Tunnel Junction.* IEEE Magnetics Letters, 2022. 13: p. 1-5.
5. Pinna, D., G. Bourianoff, and K. Everschor-Sitte, *Reservoir Computing with Random Skyrmion Textures.* Physical Review Applied, 2020. 14(5) : p. 054020.
6. Prychynenko, D., et al., *Magnetic Skyrmion as a Nonlinear Resistive Element: A Potential Building Block for Reservoir Computing.* Physical Review Applied, 2018. 9(1): p. 014034.
